# A Spatial Point Pattern Analysis in *Drosophila* Blastoderm Embryos Evaluating the Potential Inheritance of Transcriptional States

**DOI:** 10.1371/journal.pone.0060876

**Published:** 2013-04-09

**Authors:** Feng He, Jun Ma

**Affiliations:** 1 Division of Biomedical Informatics, Cincinnati Children's Research Foundation, Cincinnati, Ohio, United States of America; 2 Division of Developmental Biology, Cincinnati Children's Research Foundation, Cincinnati, Ohio, United States of America; Dartmouth College, United States of America

## Abstract

The *Drosophila* blastoderm embryo undergoes rapid cycles of nuclear division. This poses a challenge to genes that need to reliably sense the concentrations of morphogen molecules to form desired expression patterns. Here we investigate whether the transcriptional state of *hunchback* (*hb*), a target gene directly activated by the morphogenetic protein Bicoid (Bcd), exhibits properties indicative of inheritance between mitotic cycles. To achieve this, we build a dataset of *hb* transcriptional states at the resolution of individual nuclei in embryos at early cycle 14. We perform a spatial point pattern (SPP) analysis to evaluate the spatial relationships among the nuclei that have distinct numbers of *hb* gene copies undergoing active transcription in snapshots of embryos. Our statistical tests and simulation studies reveal properties of dispersed clustering for nuclei with both or neither copies of *hb* undergoing active transcription. Modeling of nuclear lineages from cycle 11 to cycle 14 suggests that these two types of nuclei can achieve spatial clustering when, and only when, the transcriptional states are allowed to propagate between mitotic cycles. Our results are consistent with the possibility where the positional information encoded by the Bcd morphogen gradient may not need to be decoded *de novo* at all mitotic cycles in the *Drosophila* blastoderm embryo.

## Introduction

Transcription is a molecular process where the enzyme RNA polymerase (RNAP) makes RNA copies from the DNA templates [1]–[3]. It is an inherently stochastic process that is reflective of the random nature of the underlying molecular events necessary for RNAPs to transcribe through a gene. These molecular events may include, for example, the remodeling of chromatin, the binding or unbinding of transcription factors and the loading or clearance of RNAPs [4]–[8]. The prevailing theoretical framework on regulation of transcription considers only the “recent” biochemical events that lead to successful transcription of a gene [9]–[12]. But can a gene’s transcriptional state be also influenced by the “past” events or its “past” experiences? We raise this question because mitosis is known to abort transcription [13], [14], yet active transcription of many patterning genes becomes detectable almost immediately after a mitotic cycle in the *Drosophila* blastoderm embryo [15]–[18]. Thus the discussions about the recent and past events in our current work make an exclusive reference to mitosis.

In *Drosophila*, embryonic patterning along the anterior-posterior (AP) axis requires the morphogen gradient of Bicoid (Bcd) [19]–[22]. Bcd is a transcriptional activator that binds to the enhancers of its target genes such as *hunchback* (*hb*) and activates their transcription [5], [9], [17], [23]. The mechanisms of sensing the concentrations of morphogen molecules by a gene or cell are crucial to our knowledge of how the positional information provided by a morphogen gradient is decoded during development [24]–[26]. Theoretical studies suggest that there are fundamental limits to the time period necessary for a cell or gene to accurately “sense” or “read” the differences in morphogen concentrations to make a response to form a desired expression boundary [27], [28]. This time period is dependent on, among other things, both the diffusion constant and the nuclear concentration of Bcd at the boundary position. Based on available measurements, the time it takes the *hb* gene to accurately read the nuclear Bcd concentration and form a *de novo* expression boundary has been calculated to be on the order of tens of minutes or longer [17], [29], [30]. This is in contrast to the observation that active transcription (at and beyond the boundary) of *hb* gene copies can take place almost immediately after mitosis, on the order of less than a minute [17], [18]. To reconcile these conflicting properties of the early *Drosophila* embryos, a memorization mechanism of an unknown nature has been proposed, where the *hb* transcriptional state of a nucleus can be influenced by the state of its parent nucleus [17]. This hypothesis provides a rational explanation for how the positional information provided by the Bcd gradient can be decoded to form reliable and precise boundaries of its target genes in early embryos that are undergoing rapid mitotic cycles in roughly every 10 minutes, a time period that would not allow *de novo* decoding and boundary formation. However, due to a lack of experimental tools suitable for tracking the transcriptional states of *hb* gene copies between mitotic cycles, this important hypothesis has remained untested.

In this study, we perform a spatial point pattern (SPP) analysis to evaluate the spatial properties of *hb* transcriptional states at the resolution of individual nuclei. SPP relates to the distribution characteristics of, i.e., spatial relationships among, a series of point locations [31]. Many biological examples, such as a sheet of cells, can be reduced to a pattern of mapped points [31], [32]. Here we treat each nucleus within the monolayer of a blastoderm embryo as a point and its transcriptional state as an event. Our study suggests properties of spatial clustering for nuclei with both or neither copies that are undergoing active transcription in snapshots of embryos. Our statistical and simulation analyses are consistent with the possibility that the *hb* transcriptional states of the nuclei can be propagated (i.e., inherited) between mitotic cycles. The statistical and simulation methods used in our current study may be of general value in investigating developmental problems that require knowledge about the spatial properties of cellular and molecular decisions.

## Materials and Methods

### Experimental Dataset

Our dataset was constructed based on our FISH data detecting the nascent transcripts of *hb* in early cycle 14 wildtype embryos (*w* ^1118^) at the peak time of active transcription of *hb* gene copies [5], [18]. These nascent transcripts were detected by a 283-nucleotide probe against the *hb* intron RNA sequence. This intron is located 145 bp downstream of the transcription start site from the Bcd-responsive *hb* promoter P2. Thus, the detected signals, which were discrete fluorescent dots inside the nucleus, referred to as intron dots, capture individual copies of *hb* undergoing active transcription near the P2 promoter [2], [5], [9]. Our current dataset consists of data from 14 whole-mount embryos, which had been flattened to maximize the number of nuclei captured. In our analysis, ∼6 confocal *z*-section images at 0.5 µm intervals were taken to capture all intron dots in the nuclei in one of the flattened sheets of the nuclear monolayer. Thresholds to identify an intron dot included both signal cluster size and signal intensity, and they were optimized to minimize false positive and false negative cases and to detect a stable intron dot expression pattern along AP axis as discussed previously [5].

**Figure 1:**
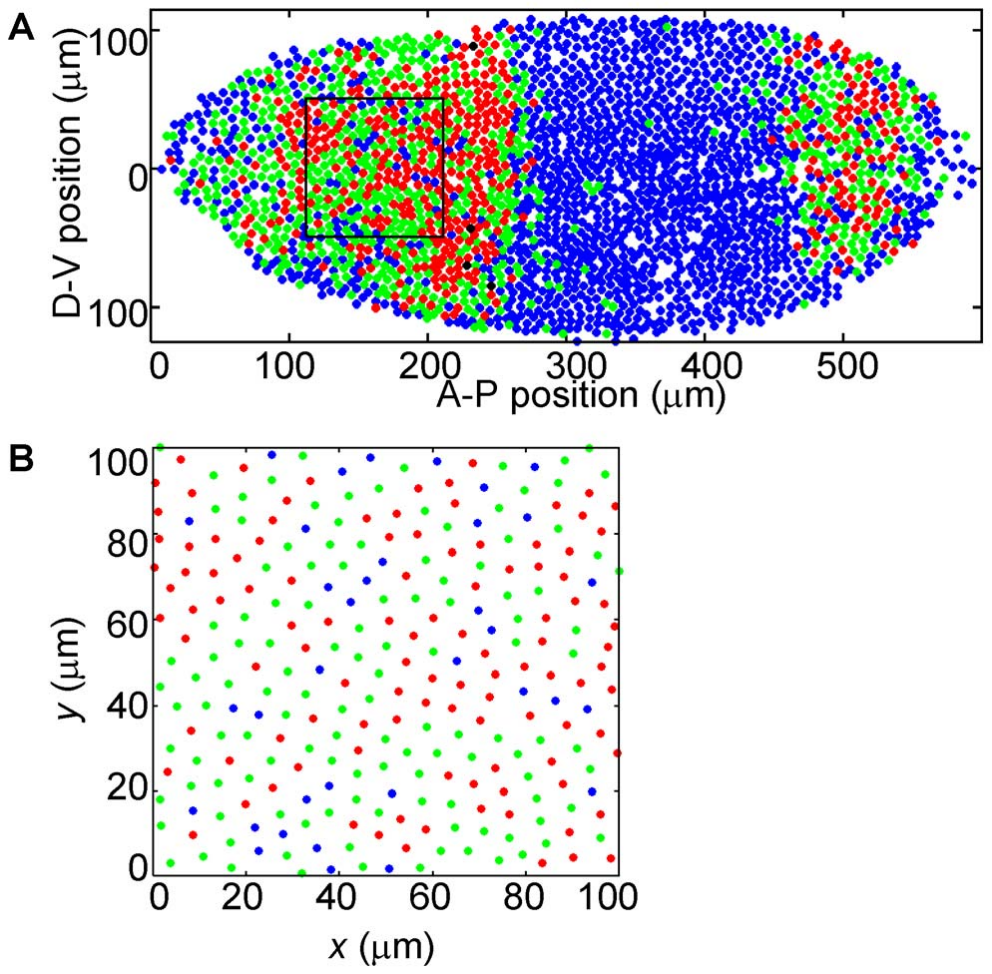
Spatial point pattern of nuclear transcriptional states. Shown is an embryo with nuclei masked according to *hb* transcriptional states, i.e., the number of *hb* intron dots detected. Blue: 0-dot nuclei; green: 1-dot nuclei; red: 2-dot nuclei; black: nuclei with over 2 dots. (B) is the cropped experimental field from the solid square in (A). See Figure S1 for locations of the experimental fields in all 14 embryos.

Each embryo was oriented with its major axis (i.e., AP axis) parallel to the *x*-axis on the image, with the anterior tip set as the origin. The center of each nucleus was identified by MATLAB and its *x*-*y* coordinates were recorded. Each image contains 3000∼4000 nuclei within the area of ∼600 µm×250 µm (Figures 1 and S1). An experimental field of 100 µm×100 µm (i.e., 130 µm to 230 µm on *x*-axis and −50 µm to 50 µm on *y*-axis) was cropped for further analysis (Figures 1 and S1). We chose this location after considering the following specific issues. 1) We avoided the edges of the embryos to minimize errors due to out-of-focus detection of nuclei. 2) We avoided the anterior tip of the embryo, where the nuclear density is known to deviate significantly from other parts of the embryo [33]. 3) We avoided the boundary of the *hb* expression domain because this boundary itself represents a spatial pattern that would interfere with our analysis. Changing the location of the cropped field by ±5 µm along either axis did not affect any of our conclusions.

### Characterizations of Nuclear Density and Inter-nuclear Distances

The overall densities of the nuclei of individual types or all types combined were calculated as *d* = *N*/*A*, where *N* denotes the number of nuclei of the type of interest within the entire field that has an area size *A* = 100×100 µm^2^ (see Results for definitions of nuclear types). The local densities of the nuclei (of either individual or combined types) were estimated at each nuclear position in the pattern by *d_i_* = *n_i_*/*πR* ^2^, where *n_i_* denotes the number of nuclei of a type within a distance *R* from the center of nucleus *i*. Here we chose *R* = 8 µm, a value greater than the diameter of a typical nucleus (∼6 µm) at this stage of embryonic development.

**Figure 2:**
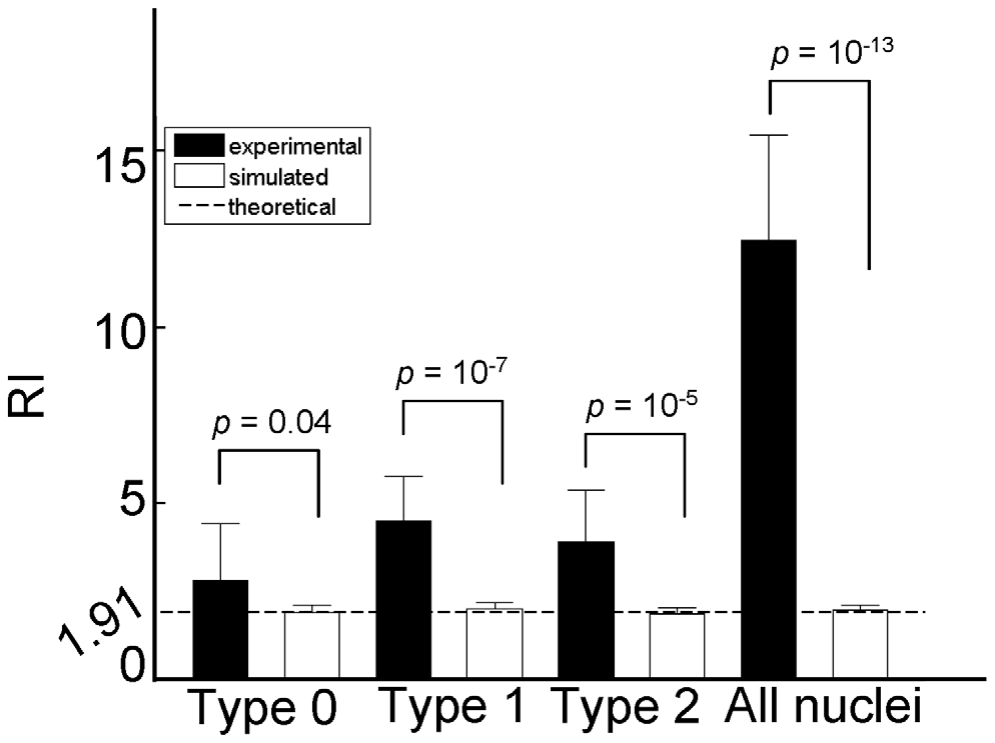
Basic features of the nuclear monolayer. Shown are regularity indexes (RI) derived by the measurement of nearest-neighbor distances. Black: results from 14 experimental fields; white: results from 14 random simulations, each of which was constrained by the total nuclear number and the proportions nuclear types observed in the corresponding experimental field. The dashed line shows the theoretical expectation of 1.91 for randomly distributed points (see also Materials and Methods). In simulations, nuclei of different types or all nuclei combined behave indistinguishable from each other or the theoretical expectation. Error bars show the standard deviations calculated from 14 experimental or simulated fields. *p*-values from paired Student’s t-tests to compare the means of experimental and simulated RI values were shown over the bars.

Center-to-center distances between all pair-wise nuclei were computed as follows. A nucleus in an experimental field was identified as the reference and the distance from that nucleus to every other nucleus in the field was measured. This procedure was repeated for every nucleus as a reference on this field and then repeated for each of the 14 experimental fields. For a given nucleus, the nearest-neighbor distance (NND) is the minimum value among its distances to all other nuclei [34]. We calculated the regularity index (RI), which was defined as the mean NND divided by its standard deviation [34]. Theoretically, for a random distribution of points in two dimensions, *RI* ≈ 1.91 [34]. This value was used as a standard against which the RI values obtained from our experimental or simulated fields were evaluated (Figure 2).

Since our experimental embryos were flattened to maximize the number of intron dots captured, we performed an analysis to assess the impact of embryo flattening on the spatial distributions of nuclei. For each blastoderm embryo (*N* = 10), we performed confocal imaging both before (i.e., free in mounting medium) and after flattening. We found that flattening an embryo led to an expansion of ∼30 µm along *x*- or *y*-axis (from 562±17 µm to 590±20 µm along *x*-axis and from 224±21 µm to 255±22 µm along *y*-axis). The distances between neighboring nuclei in the flattened sheets are increased by ∼4% relative to the 3-dimentional distances between the neighboring nuclei on the periphery of non-flattened embryos. Under both conditions, the nuclei were found to be regularly and evenly spaced, suggesting that flattening did not cause any significant localized nuclear movements within the experimental fields chosen for our analysis.

### K-function Analysis

To calculate Ripley’s K-functions [31], we binned the measured distances and counted the overall number of nuclei at each distance *r* from a reference nucleus. In effect, our exercise created annuli around the reference nucleus and measured the number of nuclei inside circles at different *r* intervals. “Edge effect” may arise for a reference nucleus within distance *r* from the boundaries of a field. We compensated this effect by surrounding the field under examination with 8 identical copies of this field (effectively forming a larger sheet that contains 9 identical “tiles”). By doing this, we were able to analyze the complete SPPs of any nuclei within the field (the center tile), including those close to the edge. We used the 2 µm *r* intervals for the annuli in our current study; we also performed analyses with increasing interval sizes and observed comparable results up to a 5 µm *r* interval size. Eq. 1 defines K-function, K(*r*), where *d* is the nuclear density.
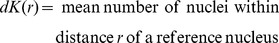
(1)


In a bivariate pattern, the nuclear densities of Type *i* and Type *j* are *d_i_* and *d_j_*, respectively. A complete description of the second-order properties of the bivariate pattern requires a consideration of all possible pairs between the opposite types. We define the bivariate K-functions K *_ij_*(*r*) by
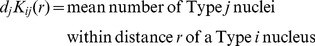
(2)


Various benchmark hypotheses when described by the K-function have well-defined expressions. In particular, the CSR hypothesis states that K(*r*) = π*r* ^2^. Independence between two opposite types has K*_ij_*(*r*) = π*r* ^2^, irrespective of the individual point patterns or the forms of K*_i_*(*r*) and K*_j_*(*r*). In our study, all K-functions were transformed to H-functions to allow visual illustration of departures from the benchmark hypotheses (see Eqs. 3–4 and the related texts in Results).

### Tests of Significance

To test statistical significance, we used a Monte Carlo approach for estimating two properties of each experimental field, 1) the K-functions and 2) the proportions of different types of nuclei that surround a nucleus of a given type. In this simulation, we used each experimental field as a “mold” for the locations of all the nuclei inside the field. Then, we randomly assigned a type to each and every nucleus while maintaining the overall proportions of the three types as observed experimentally for that mold. To evaluate significance, we performed Kolmogorov-Smirnov tests to compare experimental and simulated data.

To consider the proportions of different types of nuclei that surround a nucleus of a given type, we used the Voronoi diagram that identifies the immediate-neighboring nuclei that share an edge in a mold [35]. For each and every nucleus, we recorded the number of immediate-neighboring nuclei belonging to each of the three types in either experimental or simulated fields. For each mold, we performed 199 simulations and estimated the 95% confidence interval (CI) using the results that ranked as the top or bottom 5 simulations. In addition to the Monte Carlo approach, we also theoretically predicted the CI by treating each of the 14 experimental patterns as a draw from a probability distribution. Specifically, we assumed that, in a population of *N* nuclei, the number of a given type (*n*) follows a binomial distribution with probability *P_i_*, i.e., *n* ∼ *B*(*N*, *P_i_*). Then the 95% CI was determined by
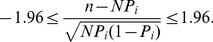
(3)The parameter *P_i_* was estimated for each nuclear type in the experimental fields.

### Modeling Nuclear Lineages and Mitotic Inheritance

To simulate the nuclear lineages, we used the nuclear positions shown in Figure 1B as the mold and randomly assigned every 8 neighboring nuclei within the mold into a clone. This clone represents the nuclei that would have been derived from a single nucleus at cycle 11 after undergoing 3 mitotic cycles. Then, we randomly divided each of the 8-nuclei clones into two 4-nuclei clones. These two clones represent the nuclei that would have been derived from the two nuclei at cycle 12 after undergoing 2 mitotic cycles. Finally, we randomly divided each of the 4-nuclei clones into two 2-nuclei clones, which represent the nuclei that would have been derived from two nuclei at cycle 13 after undergoing one mitotic cycle. Effectively, this exercise led to the generation of nuclear lineages from cycle 11 to 14 without specifically modeling or knowledge of the nuclear positions and movements prior to cycle 14.

To simulate the spatial patterns of transcriptional states of nuclei in blastoderm embryos, we assumed, based on reported experimental data [17], that the probability of *hb* gene copies undergoing active transcription (referred to as the transcribing probability thereafter) at cycles 11 to 14 is 90%, 90%, 50% and 50%, respectively. In simulations where there is no inheritance, we assumed the number of active copies in each nucleus as a random draw from a binomial distribution, which is a function of transcribing probability at each cycle. In simulations where inheritance was allowed, the two daughter nuclei (at each new cycle) were assigned to exist as the same type as their parental nucleus. Upon entering the cycle 13 interphase, active copies were randomly switched off to achieve the experimentally observed reduction in the transcribing probability. We performed 99 simulations for each of the two scenarios.

## Results

### The SPP Dataset and Basic Features of the Nuclear Monolayer

To analyze the spatial point pattern (SPP) of nuclei with distinct *hb* transcriptional states, we constructed a dataset from images of 14 *Drosophila* embryos at early nuclear cycle 14 (see Materials and Methods). These images represent snapshots capturing robust *hb* transcription at the resolution of individual copies of the gene inside the nuclei of the embryo [5]. Here an intronic probe for *hb* was used in FISH to detect its nascent transcripts in whole-mount embryos. These nascent transcripts were detectable as distinct fluorescent dots, referred to as intron dots, each representing, in the snapshot, a copy of *hb* undergoing active transcription near the Bcd-responsive P2 promoter (see Materials and Methods). Our dataset includes the identities and locations of the nuclei containing 0, 1 or 2 intron dots (Figure 1A blue, green and red). We define all nuclei with a same number of intron dots as being one type. Thus there are three types of nuclei: Type-0, Type-1 and Type-2 (a minimal number of nuclei with erroneously more than 2 dots detected were also treated as Type-2 nuclei; Figure 1A black). The fractions of the three types of nuclei in our current dataset (see Table S1 for nuclear counts in individual embryos) are well explained by a binomial distribution, which argues against bistability of transcriptional states [9].

It has been shown that Bcd induces *hb* transcription in a highly cooperative manner [5]. This cooperative action [36], [37] effectively divides an embryo into two broad domains along the anterior-posterior (AP) axis [5], [17], [38]. Here we refer to the anterior portion of the embryo with Bcd-dependent active *hb* transcription as the transcribing domain, whereas the posterior portion lacking Bcd-dependent active *hb* transcription as the non-transcribing domain. In our current study, we focus exclusively on the transcribing domain. Here our goal is not to analyze directly how the two domains are established (i.e., boundary formation as a function of the Bcd input in a threshold response) but, rather, within the transcribing domain, how the nuclei with distinct *hb* transcriptional states are distributed spatially. To facilitate our analysis, we cropped the images to obtain areas (i.e., experimental fields) of an identical size within the transcribing domains of individual embryos (see also Materials and Methods and Figure 1B). Since visual inspections (Figure 1B) are not adequate for extracting and describing the characteristics of the spatial relationships among the nuclei of a given type, we approached this problem through the use of statistical methods.

Several methods have been developed to analyze SPP data [31], [32]. In our study, we used first-order statistics to characterize the basic features of the nuclear monolayer in cycle-14 embryos with regard to nuclear density and variations. Here the experimental fields have a mean nuclear density (*d*) of 0.024 nuclei/µm^2^ (averaged among different embryos) and a standard deviation (SD) of 0.002 nuclei/µm^2^. The overall densities of Types-0, -1 and -2 nuclei in the fields are 0.004±0.001, 0.011±0.002 and 0.009±0.002 nuclei/µm^2^, respectively. Previous studies have shown that an embryo at early cycle 14 interphase has relatively uniform nuclear density throughout, with a dominant exception at its terminal regions [33], [39], [40]. In our chosen experimental fields (see Materials and Methods), the local densities of all nuclei, Types-0, -1 and -2 nuclei are 0.026±0.003, 0.013±0.005, 0.013±0.004 and 0.015±0.004 nuclei/µm^2^, respectively (all values are averaged among different nuclear positions with SD shown). These results show that, consistent with previous reports, our experimental fields, which were chosen to be devoid of the terminal regions of the embryo (see Materials and Methods and Figure S1), do not exhibit major variations in local nuclear densities.

To further evaluate the basic features of the nuclear monolayer, we calculated the regularity index (RI). RI is defined as the ratio of the mean of the nearest-neighbor distance (NND) to its standard deviation. It measures the uniformity of NND, providing a widely-used criterion for evaluating spatial regularity [41], [42] (see also Materials and Methods). NND calculation does not take into account the actual size of the nucleus. Thus the relatively uniform size and spacing of the nuclei in the monolayer are features of spatial regularity of the nuclei themselves under the framework of the RI analysis (see also below for further support to this notion). Figure 2 plots the RI values of the three types of nuclei and all types combined (solid bars; RI = 2.81±1.61, 4.49±1.29, 3.90±1.48 and 12.49±2.96, for Types-0, 1, 2, and all types, respectively). To statistically evaluate spatial regularity, we generated a random distribution of nuclei within a two-dimensional field and calculated RI values for the simulated data. Here, both the field size and nuclear number, but not nuclear positions, were constrained by our experimental measurements of an experimental field. This analysis was performed for each of the 14 experimental fields. We found that the experimentally measured RI values (Figure 2 solid bars) are significantly higher than their corresponding values derived from random simulations (Figure 2 white bars; RI = 1.89±0.14, 2.01±0.17, 1.87±0.09 and 1.97±0.11, respectively; *p* = 0.04, 10^−7^, 10^−5^ and 10^−13^, respectively). In each case, these values are also higher than the theoretical value calculated for random spatial distributions of points (see Materials and Methods for details about this theoretical value and simulations). These results confirm quantitatively that the nuclei themselves within our experimental fields of the nuclear monolayer have regular spacing and uniform size.

### Type-0 and Type-2 Nuclear Patterns are Formed Non-randomly

To investigate the spatial distributions of the nuclei, we conducted an analysis using second-order statistics. Unlike the distance-based NND method, second-order statistics describes the characteristics of the point distributions as a function of area size. Here we used Ripley’s K-function, which has been extensively employed in analyzing spatial patterns in biology [43], [44]. The K-function analysis uses all inter-nuclei distances to evaluate a spatial point pattern. It measures the number of events that occur inside the area of a circle with a radius *r* from a reference event. In essence, a circle of a specific radius *r* is passed over a pattern, and the number of events that occur inside this circle is counted. This counting is repeated at different radii for this nucleus and then repeated for all nuclei. Thus, the K-function is presented as a plot against *r*. Note that the definition of K-function has been arranged such that it is only a function of *r*. It is independent of the nuclear density *d* of the experimental fields (see Materials and Methods). Since the K-function analysis is conducted at multiple radii, it can reveal a pattern’s characteristics that are dependent on the scales of neighborhood size being evaluated. This particular feature of K-function analysis circumvents the limitations of the NND method and, thus, can reveal deeper information about spatial patterns.

In our study, all K-functions were transformed to.
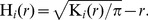
(4)


Subtraction of *r* facilitates a visual presentation illustrating departures from complete spatial randomness (CSR). In this analysis, CSR is the null hypothesis stating that the positioning of the nuclei of a given type (the events) within the experimental fields follows a homogeneous Poisson process. Thus violations of this null hypothesis may arise from major variations in the local density of events. In Eq. 4, a positive H*_i_*(*r*) value means that, on average, there are more nuclei of Type *i* inside the area of a circle with a radius *r* than expected of CSR. Thus, a positive H*_i_*(*r*) value indicates spatial clustering when evaluated at *r*. Conversely, a negative H*_i_*(*r*) value indicates repulsion among the nuclei at *r*.

**Figure 3:**
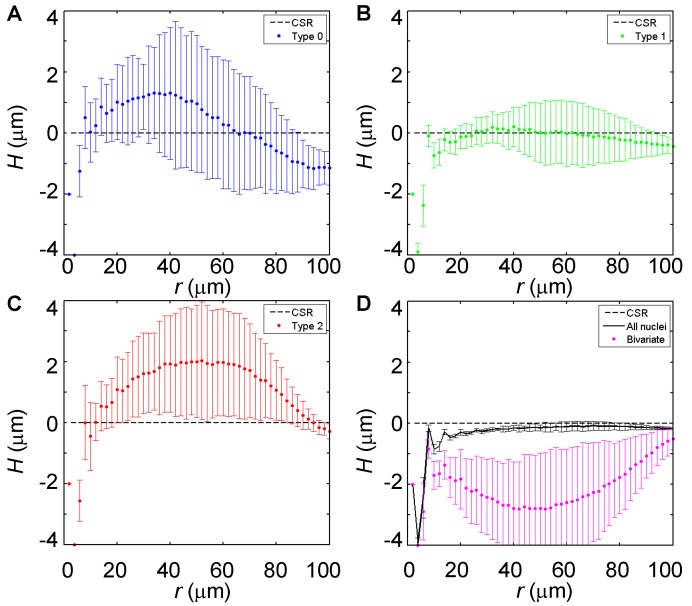
H-function analysis of experimental fields. Shown are the profiles of H-functions with respect of the distance *r* for Type-0 nuclei (A), Type-1 nuclei (B), Type-2 nuclei (C), Type-0/Type-2 bivariate (purple dots in D) and all nuclei combined (solid lines in D). The dashed line shows the theoretical expectation of complete spatial randomness (CSR; see also Materials and Methods). Error bars show the standard deviations calculated from 14 experimental fields.

Figure 3 shows the H-functions of different types of nuclei plotted against *r*. On scales approaching the nuclear diameter (4∼8 µm at early cycle 14), the H-functions of the nuclei of each individual type or all types combined are all negative, indicative of a repulsive effect among these nuclei. This result is simply reflective of the basic feature of the nuclear monolayer discussed above, namely, the relatively uniform size and spacing of the nuclei themselves. However, on scales exceeding the typical size of the nucleus, the H-functions of Types-0 and -2 nuclei both become positive (Figure 3A and C). These results show that the nuclei of these two types have properties of spatial clustering. In our analysis, Type-1 nuclei exhibit no significant difference from CSR (Figure 3B).

### Type-0 and Type-2 Nuclei are Distributed as Dispersed Clusters

If Types-0 and -2 nuclei are indeed spatially clustered, there is a possibility that the nuclei of these two opposite types may exhibit properties of mutual repulsion. To evaluate this possibility, we calculated the bivariate H-function (Eq. 5). Here we used a nucleus of one type as a reference point (i.e., Type *i*) and counted the number of nuclei of the opposite type (i.e., Type *j*, *i* ≠ *j*) that fall within the area of a circle with radius *r*.
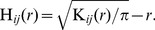
(5)


For bivariate point patterns, where patterns of two opposite events (i.e., the nuclei of two different types) are considered in relation to each other, the null hypothesis is independence. Under this null hypothesis, H*_ij_*(*r*) = 0. H*_ij_*(*r*) >0 indicates that, on average, there are more Type-*j* nuclei (than expected of independence) inside the area of a circle with a radius *r* from a Type-*i* nucleus. This suggests an attraction between the nuclei of the opposite types. Conversely, H*_ij_*(*r*) <0 suggests repulsion between the nuclei of the opposite types. Either attraction or repulsion would reject the null hypothesis stating that these two opposite events are determined independently of each other. Based on the bivariate H-function analysis (Figure 3D), Types-0 and -2 nuclei exhibit properties of mutual repulsion (Figure 3D purple). Using the same approach, the null hypothesis cannot be rejected when the two opposite events are Type-0 and Type-1 nuclei or are Type-1 and Type-2 nuclei (data not shown).

**Figure 4:**
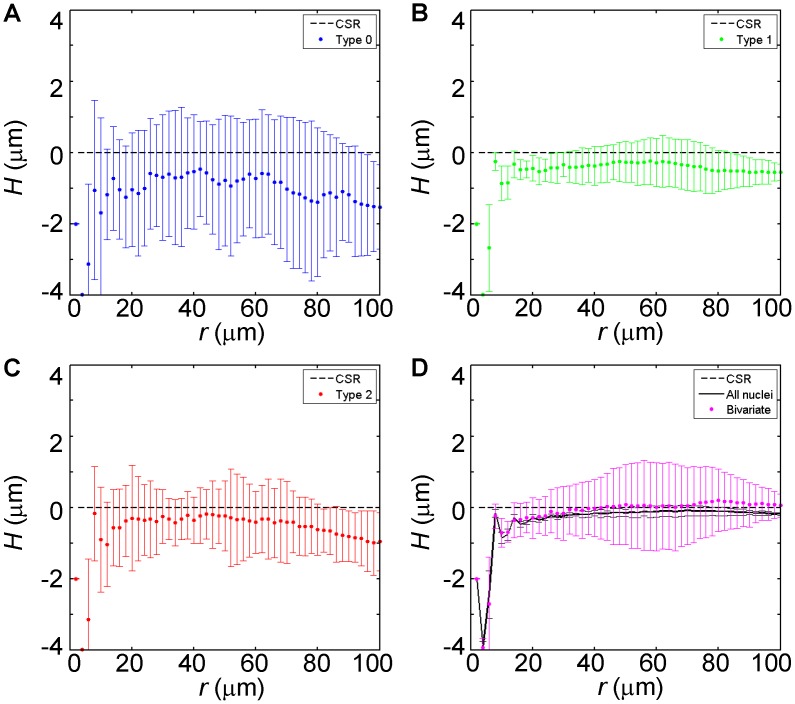
H-function analysis of simulated fields. Shown are results of an analysis that is identical to that of Figure 3, but simulated fields are used here. Unlike simulations shown in Figure 2, the simulations performed here were based on the molds derived from the experimental fields, with both the locations of the nuclei and the proportions of the nuclear types constrained by experimental observations. Note that the profiles of individual nuclear types are all below 0. This is expected because the nuclei of a given type are spatially mixed with the nuclei of other types when randomly distributed and thus the nuclei of a given type always exhibit a self-repelling SPP. Moreover, because the proportion of Type-0 nuclei is the smallest, the H-function profile of this type is the most negative among all three types. Also note that the profiles do not converge to zero at *r* = 100 µm, which is the boundary of the reference nuclei to compute distance, but not the limit of a complete SPP (see also Materials and Methods).

To extend the evaluation and understanding of our experimental data, we generated randomly simulated data based on each of the “molds” derived from the 14 experimental fields (see Materials and Methods). Figure 4 plots the H-function profiles of the simulated data. Here we used Kolmogorov-Smirnov tests to further evaluate the differences between the H-functions of the experimental and simulated data (shown in Figures 3 and 4, respectively). For each pattern, we determined the range of *r*, within which the H(*r*) differences are significant (*p*<0.05). The *r* ranges for Type-0 analysis, Type-2 analysis and Type-0/Type-2 bivariate analysis are 6∼44 µm, 12∼80 µm and 6∼100 µm. They document that the experimental and simulated data differ in a consistent manner within broad *r* ranges, which contrasts with the narrow *r* range of 30∼34 µm for Type-1 analysis. Together these results provide further support to the observation that, in experimental fields, Types-0 and -2 nuclei are spatially clustered and these two types of nuclei exhibit mutual repulsion.

### Distributions of Different Types of Nuclei in Spatial Relations to Type-2 Nuclei

**Figure 5:**
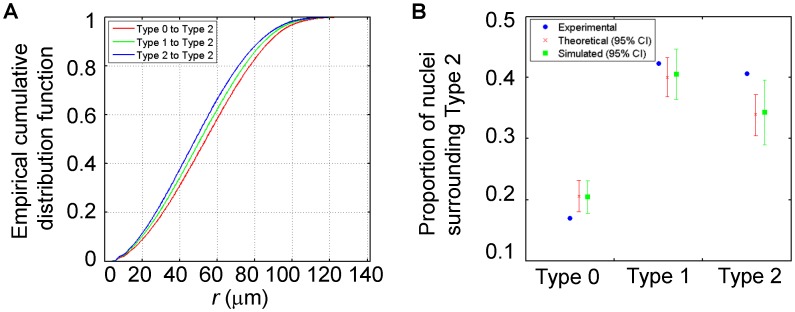
Distributions of different types of nuclei in relations to a Type-2 nucleus. Shown are the empirical cumulative distribution function plots of the distances from a Type-2 nucleus to another nucleus of any given type (A), and the proportions of nuclei of different types that immediately surround a Type-2 nucleus (B). Here the analysis was performed on the entire dataset pooled from 14 experimental fields; see Figure S2 for results performed on individual embryos as in panel (B).

If Type-2 nuclei in our experimental fields form local clusters and, conversely, Type-0 and Type-2 nuclei exhibit mutual repulsion, there is a corollary that can be tested. It states that, from a given Type-2 nucleus, it would take a shorter distance to reach another Type-2 nucleus than to reach a Type-0 nucleus in our experimental fields. To test this corollary through the use of an independent method (other than the use of Ripley’s K-function), we plot the empirical cumulative distribution functions for the distances between a Type-2 nucleus and another nucleus of any type. Figure 5A shows that pairs of Type-2 nuclei are more likely to be closer (blue) than pairs of nuclei of different types (green and red). Kolmogorov-Smirnov tests suggest that, at the 1% significance level, all these three distributions are different.

To further confirm the clustering property of Type-2 nuclei observed by Ripley’s K-function, we performed another analysis. If Type-2 nuclei in our experimental fields do form clusters that are not random in nature, we would expect to observe a difference between our experimental data (blue in Figures 5B and S2) and randomly simulated data with regard to the distributions of the types of nuclei immediately surrounding a Type-2 nucleus. We calculated the envelopes of the 95% confidence interval (CI) from either a theoretical model (red in Figures 5B and S2) or random simulations (green in Figures 5B and S2). The theoretical model and random simulations are detailed in Materials and Methods. Among nuclei surrounding a Type-2 nucleus, the proportions of both Type-0 and Type-2 nuclei lie outside the 95% CI, with the former being smaller and latter being greater than random. Equivalent results were also obtained for the proportions of different types of nuclei that surround a Type-0 nucleus, but not when evaluating the neighborhood of a Type-1 nucleus (data not shown). Together these findings suggest that, in early cycle 14 embryos, transcriptional states of *hb* genes are likely to be correlated among immediate-neighboring nuclei.

### A Nuclear Lineage Model Evaluating the Outcome of Inheritance of Transcriptional States

A simplified, idealized model of inheritance or memorization can be stated as follows, although the actual biological system is likely much more complex. A nucleus derived from a parental nucleus upon a mitotic cycle adopts both the parental location (i.e., either at or immediately adjacent to this location) and the parental state of transcription (i.e., of the same type). To determine whether this simple model may be able to capture some of the key features of the experimentally observed spatial distributions of the nuclei, we performed simulations using a series of nuclear lineage patterns from cycle 11 to cycle 14. Using the positions of experimentally observed cycle-14 nuclei (the mold) shown in Figure 1B, we assigned randomly all nuclei into 8-neighbor lineage clusters (see Materials and Methods and also Figure S3). Based on experimental observations [17], we used a 90% transcribing probability for *hb* gene copies at cycles 11 and 12, and 50% at cycles 13 and 14. This decrease in transcribing probability was shown to coincide with a decay of the maternal Hb, marking a transition from a virtually uniform (also referred to as “synchronous”) to stochastic *hb* transcription [17]. The number distributions of the experimentally observed three types of nuclei within the *hb* transcribing domain are well described by a binomial function irrespective of the overall transcribing probability [9], [17].

**Figure 6:**
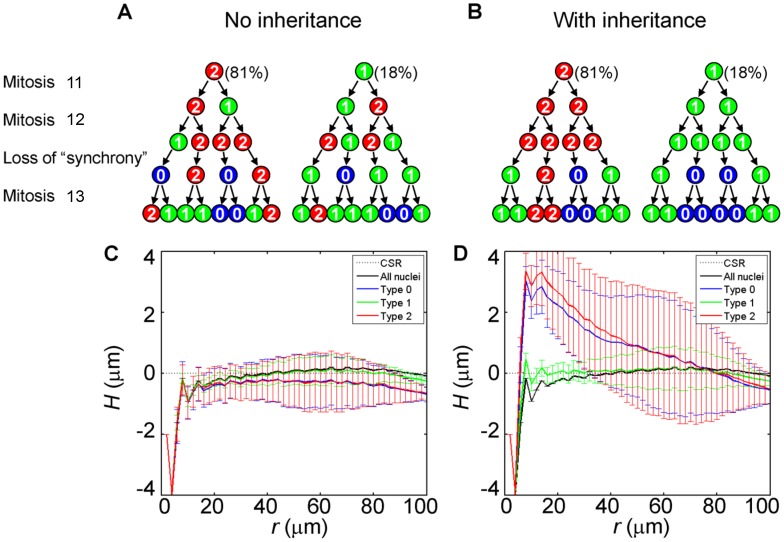
Nuclear lineage simulations with and without transcriptional inheritance. Shown are simulated examples (A and B) and the H-function profiles (C and D) of the nuclear types with (B and D) or without (A and C) inheritance of transcriptional states. These depicted examples are for illustrative purposes by showing the simulated transcription state lineages with nuclei aligned in one dimension; see Figure S3 for simulated examples of 8-nuclei lineage clusters arranged in two-dimensional fields. For the initial conditions at cycle 11, the probability of *hb* busting is set at a high level as observed experimentally (see also Materials and Methods). With an overall transcribing probability of 90%, the majority of nuclei are Type 2 (81%), with Type-1 being 18% and Type-0 being 1% (not shown in the diagrams that are for illustrative purposes only). At cycle 13, a binomial process is implemented in our simulation that randomly shuts off active *hb* copies so that the overall transcribing probability becomes 50% (with Types-0, -1 and -2 being 25%, 50% and 25%, respectively). The type of each nucleus in (A) is assigned by the binomial random number generator of MATLAB.

We performed 99 simulations either with or without inheritance. When inheritance of transcriptional states between mitotic cycles was not implemented, all *hb* gene copies were allowed to undergo active transcription in a random and independent manner. In this case, the number of active copies in each nucleus followed a binomial distribution, which was a function of transcribing probability at each cycle (Figure 6A). When inheritance was implemented, the two daughter nuclei (at each new cycle) were assigned to exist as the same type as their parental nucleus. Active copies were randomly switched off to achieve the experimentally observed reduction in transcribing probability at cycle 13 (Figure 6B). To evaluate the spatial distributions of the simulated data, we computed the H-function profiles. Without inheritance, Types-0 and -2 nuclei behave closely to CSR (Figure 6C; see also Figure S4A for one simulated pattern). With inheritance, the H-functions of these two types of nuclei become positive values within the *r* range of 6∼78 µm. These simulated properties are robust to the choices of transcribing probabilities: reducing the overall transcribing probability at cycle 11 and 12 from 90% to 60% or varying the probability at cycles 13 and 14 between 40% and 60% did not alter the outcomes (data not shown). We note that the simulated clustering (Figure 6D; see also Figure S4B) is tighter than observed in the experimental data (Figure 3A and 3C), with both greater H(*r*) values and a narrower *r* range, especially for Type-2 nuclei. This difference suggests that the actual biological system may not follow the strict inheritance as implemented in our idealized model (see below for further discussions). This difference may also be contributed by additional underlying differences between the actual biological system and our simulated system. For example, the 8-nuclear lineage clusters used in our simulations (3.4±0.5% EL; see also Figure S3A) are tighter than the experimentally observed clusters (4.8±1% EL, [17]).

## Discussion

Understanding how the spatial information is encoded by morphogen gradients and decoded by cells is of fundamental importance to developmental biology and biophysics [45]–[48]. Recent studies, fueled by both technological and conceptual advances, have uncovered important insights into this problem. Most of the existing studies have focused primarily and directly on how the boundaries of gene expression patterns are established. Unlike these studies, our current study focuses on a distinct, but related (see below), aspect of transcriptional responses to morphogen action. Specifically, we investigate the spatial distributions of nuclei exclusively within the *hb* transcribing domain of *Drosophila* embryos. Our previous studies of *hb* transcriptional states were based on grouping of nuclei into bins along the AP axis to permit direct evaluations of the threshold response to the Bcd gradient on an embryonic scale [5], [9]. A bin of 2% EL contains on average <2 nuclei along the AP axis. Meanwhile, the nuclear lineage from cycle 11 to 14 has an average width of 4.8±1% EL [17]. Thus the binning would arbitrarily break the territories of any nuclear lineages and, consequently, lead to a loss of the full spatial information on a finer scale. A bin within in the transcribing domain can cross >10 different territories [17], effectively eliminating (through averaging within the bin) any nucleus-to-nucleus correlations that may exist within individual territories. This feature is suitable for specifically evaluating transcriptional states along the AP axis. In contrast to the binning method, our current study preserves the native spatial information about the nuclei in the monolayer within the *hb* transcribing domain. This permits the extraction of the features about the spatial relationships among the nuclei with regard to their *hb* transcriptional states.

Our experimental results show that, due to the nearly uniform size of the nucleus and the tight packing against one another in the blastoderm embryo, the nuclei themselves are regularly spaced when evaluated on a scale at or below the mean nuclear diameter (Figure 2). However, on larger scales, nuclei with both or neither *hb* copies undergoing active transcription (i.e., Type-2 and Type-0) exhibit properties of spatial clustering. These results can be explained by a model where transcriptional states of the nuclei are inherited between mitotic cycles (Figure 6D). Thus, although our current analysis focuses exclusively on properties within the transcribing domain, it illuminates on the potential inheritance of transcriptional states, an issue fundamental to how gene expression boundaries are formed in response to the Bcd concentration gradient during development (see Introduction). Our findings are supportive of a hypothesis that active *hb* transcription may not depend on *de novo* sensing–at all mitotic cycles–of the nuclear Bcd concentration to form an expression boundary [17].

In our analysis of the experimental data, nuclei with a single active *hb* copy showed little or no evidence of spatial clustering. We currently do not fully understand why Type-1 nuclei behave differently from Type-0 and Type-2 nuclei. We note that our simulations based on the experimentally obtained molds also revealed a lack of spatial clustering of Type-1 nuclei (Figure 6D). Type-1 and Type-2 nuclei are similar in the sense that they both have at least one transcribing *hb* copy and are thus transcriptionally “active” [4], [17]. But they are vastly different from a statistical point of view. The binomial distribution of the observed probability of the three types of nuclei within the transcribing domain dictates that Type-1 nuclei are the largest fraction at cycle 14 (when the overall transcribing probability is 50%; see Figure 6 legend for expected fractions of the three types based on binomial distribution). Thus, among all three types, Type-1 nuclei have the highest probability of being involved in potential type switches. These switches would likely take place at random locations in the embryo, which may further contribute to both the observed lack of clustering of Type-1 nuclei and the relatively loose clustering of the other two types.

We note that, since our statistical analyses cannot draw direct conclusions about causal relationships, experimental validations of inheritance of the transcriptional states for Bcd target genes such as *hb* will await technological advances in the future. We also note that there are documented precedents for inheritance of transcriptional states between mitotic cycles. For example, studies of the transcriptional states of the globin genes within clonally-expanded populations of mouse cells suggested that stochastic decisions can be inherited and maintained between mitotic cycles [49]. Positional effect variegation (PEV) is another example of mitotic inheritance where the chromatin structure is known to play a role [50], [51]. In neither case, to our knowledge, has the timing of the onset of active transcription of individual gene copies upon exiting the mitosis been carefully evaluated. While these and other examples of inheritance may be related to the inheritance that we investigate here, the timing of the onset of active transcription after mitosis in our case is very quick, which is on scales that are likely measured by seconds or tens of seconds as opposed to minutes or tens of minutes [4], [17], [18]. It has been proposed that promoters with stalled RNAP can respond to induction more quickly [2], [52]. There is evidence that the *hb* P2 promoter, similar to promoters of many other patterning genes [53], contains stalled RNAP [54]. If the stalled RNAP were responsible for the inheritance that we investigate here, how would it arrive at the gene promoter so quickly (after mitosis) in the first place? Stalled RNAP probably could not have survived the mitotic process. It has been proposed that transcription takes place at discrete nuclear locations referred to as the transcription factories [55], [56]. Could the actively-transcribing *hb* copies, i.e., the intron dots, take residence in these factories? Could the Bcd molecules that are enriched at the intron dot locations [5], and/or maternal Hb which plays a role in increasing the transcribing probability of *hb* in earlier cycles [17], also take residence in these factories? It remains a significant future challenge to understand whether and in what fashion these factories might contribute, if at all, to the memorization or inheritance of transcriptional states between mitotic cycles. Despite unresolved questions, our current study represents an important step toward understanding both the decoding of positional information during development and the fundamental transcription process.

## Supporting Information

Figure S1 **Location of the experimental field within the contours of all 14 embryos.** The dots in different colors represent the nuclear positions at the outer edges of 14 embryos; the solid box represents the field, where our analyses were performed.
(TIF)

Figure S2 **Estimation of proportions of nuclei surrounding a Type-2 nucleus for individual embryos.** Same as Figure 5B but for individual embryos.
(TIFF)

Figure S3 **Simulated nuclear lineages.** Shown are nuclear lineages generated from one random simulation based on the mold shown in Figure 1B. We performed 99 simulations in total. In each panel, neighboring nuclear lineages are distinguished by different colors. (A-C) represent 8-nuclei lineages from cycle 11 to cycle 14, 4-nuclei lineages from cycle 12 to cycle 14, and 2-nuclei lineages from cycle 13 to cycle 14, respectively. See Materials and Methods for details.
(TIFF)

Figure S4 **Simulated nuclear patterns of transcriptional states.** Shown are examples of simulated fields exhibiting the transcriptional states of the nuclei, with (B) or without (A) inheritance. These two simulations were based on the exact nuclear lineage assignments shown in Figure S1.
(TIFF)

Table S1 **Nuclear numbers of each type in the experimental fields of all 14 embryos.** For each type of nuclei, the mean and the standard deviation among 14 embryos are given in the last column.
(DOC)
