# Supplementary figures and images for: A Spatial Point Pattern Analysis in Drosophila Blastoderm Embryos Evaluating the Potential Inheritance of Transcriptional States
Source: PLoS One. 2013 Apr 9;8(4):e60876. doi: 10.1371/journal.pone.0060876 (PMC3621909; doi:10.1371/journal.pone.0060876)

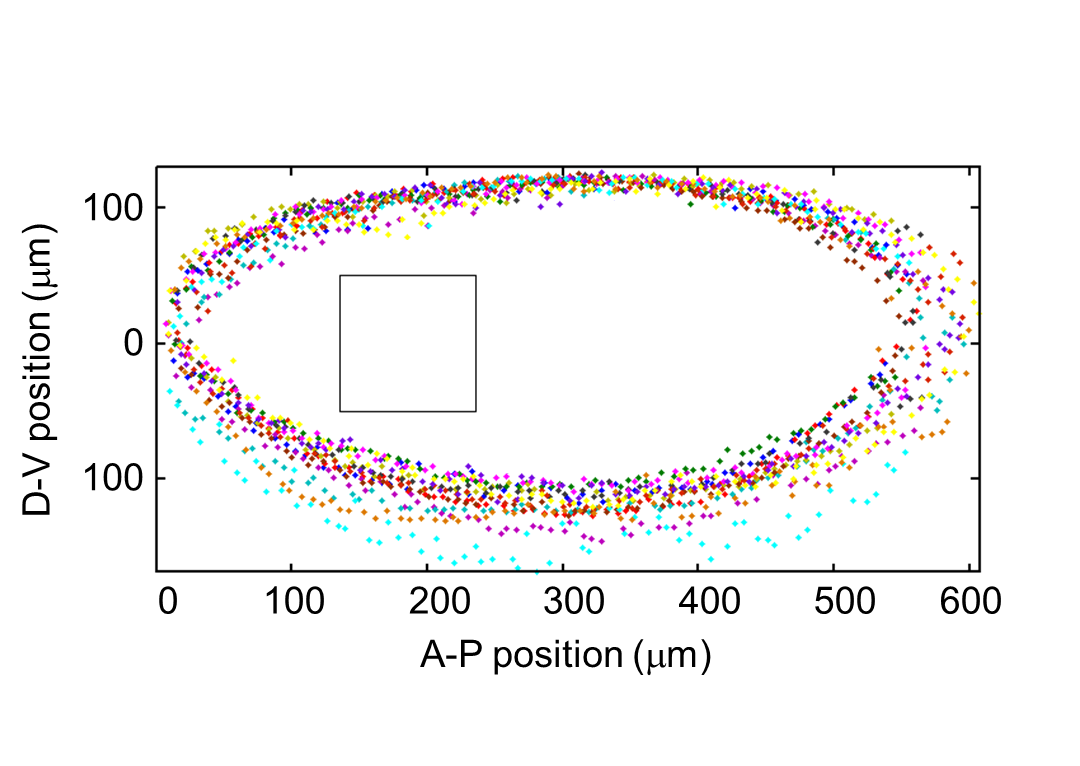

Supplement: Figure S1 — Location of the experimental field within the contours of all 14 embryos. The dots in different colors represent the nuclear positions at the outer edges of 14 embryos; the solid box represents the field, where our analyses were performed. (TIF) [file pone.0060876.s001.tif]

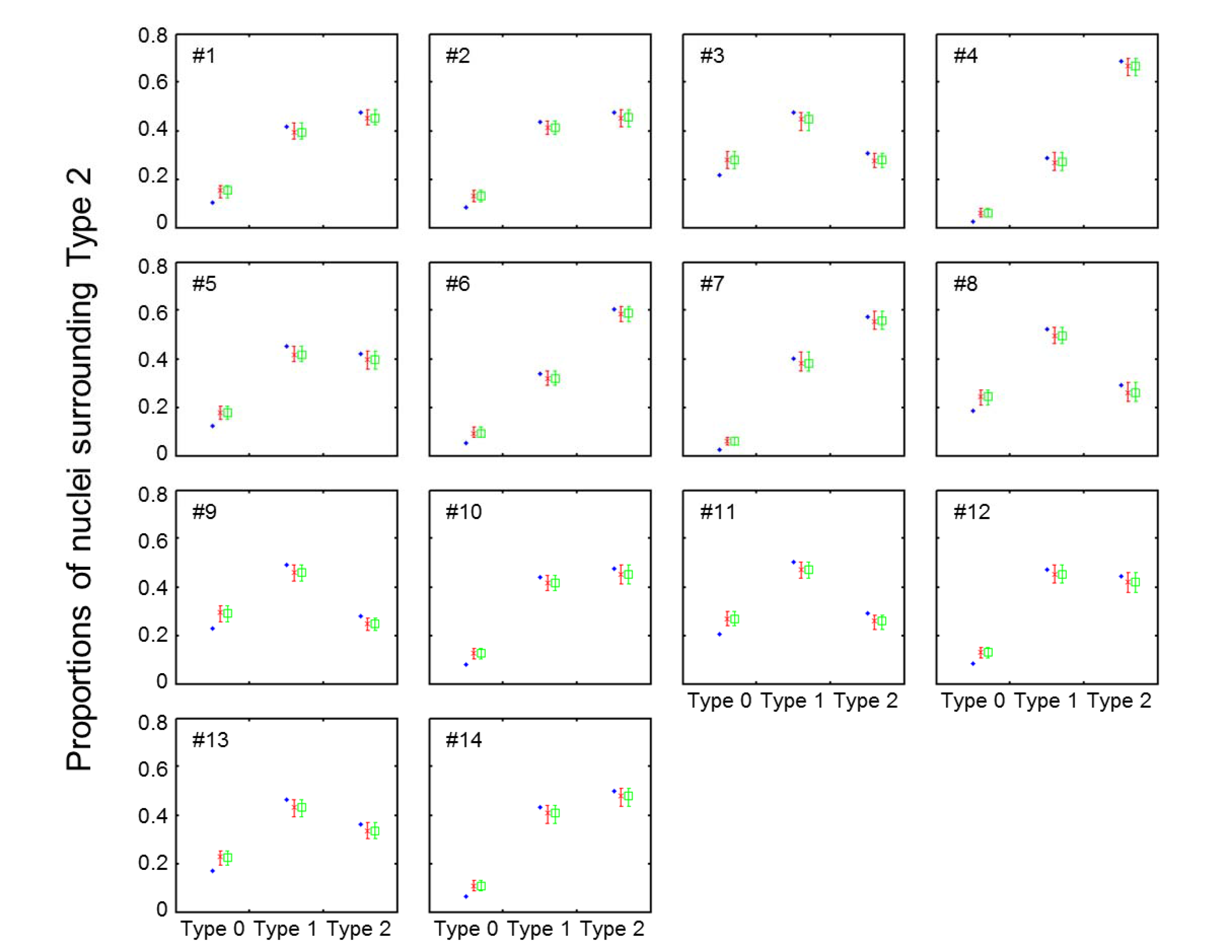

Supplement: Figure S2 — Estimation of proportions of nuclei surrounding a Type-2 nucleus for individual embryos. Same as Figure 5B but for individual embryos. (TIFF) [file pone.0060876.s002.tiff]

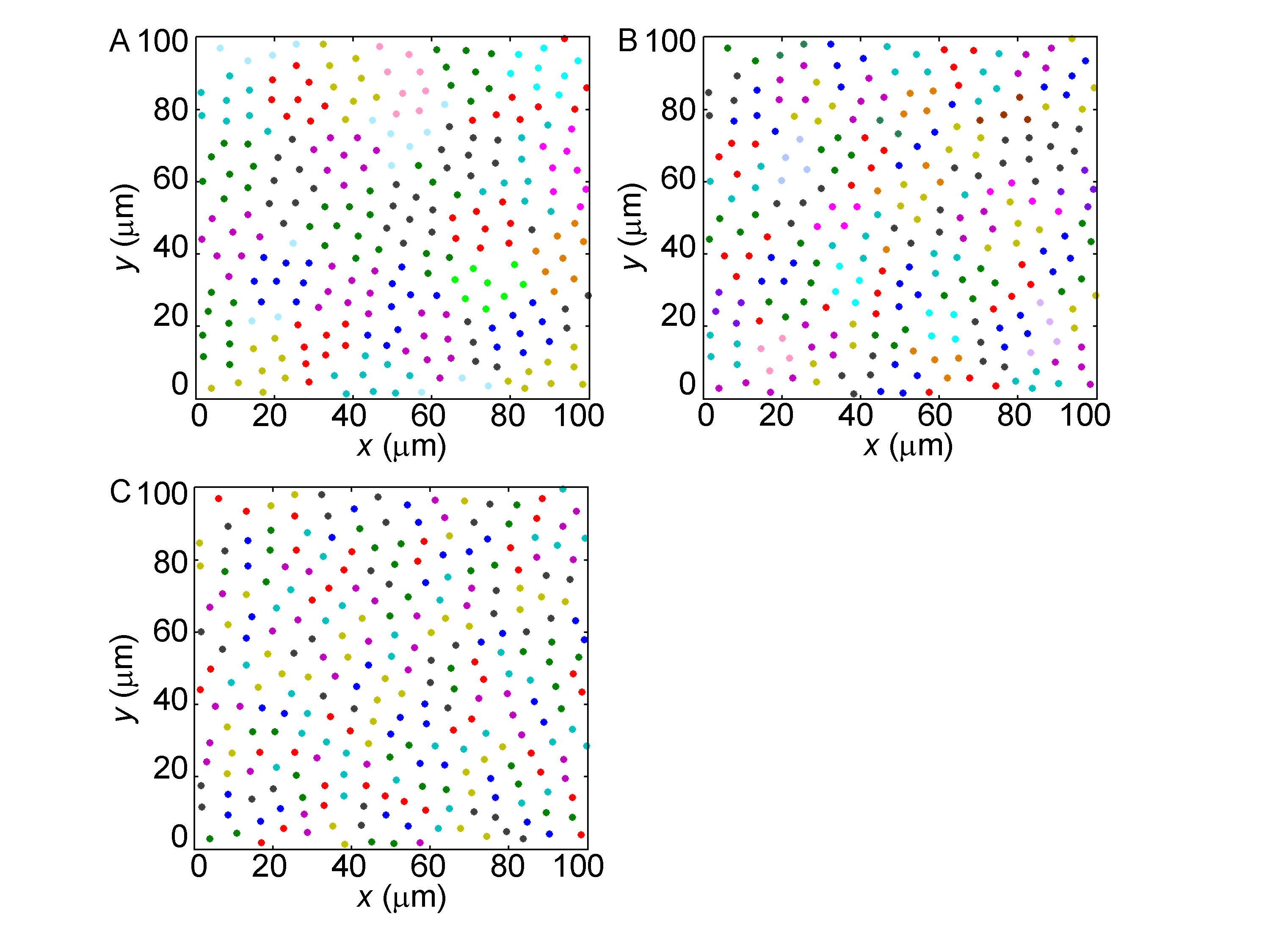

Supplement: Figure S3 — Simulated nuclear lineages. Shown are nuclear lineages generated from one random simulation based on the mold shown in Figure 1B. We performed 99 simulations in total. In each panel, neighboring nuclear lineages are distinguished by different colors. (A-C) represent 8-nuclei lineages from cycle 11 to cycle 14, 4-nuclei lineages from cycle 12 to cycle 14, and 2-nuclei lineages from cycle 13 to cycle 14, respectively. See Materials and Methods for details. (TIFF) [file pone.0060876.s003.tiff]

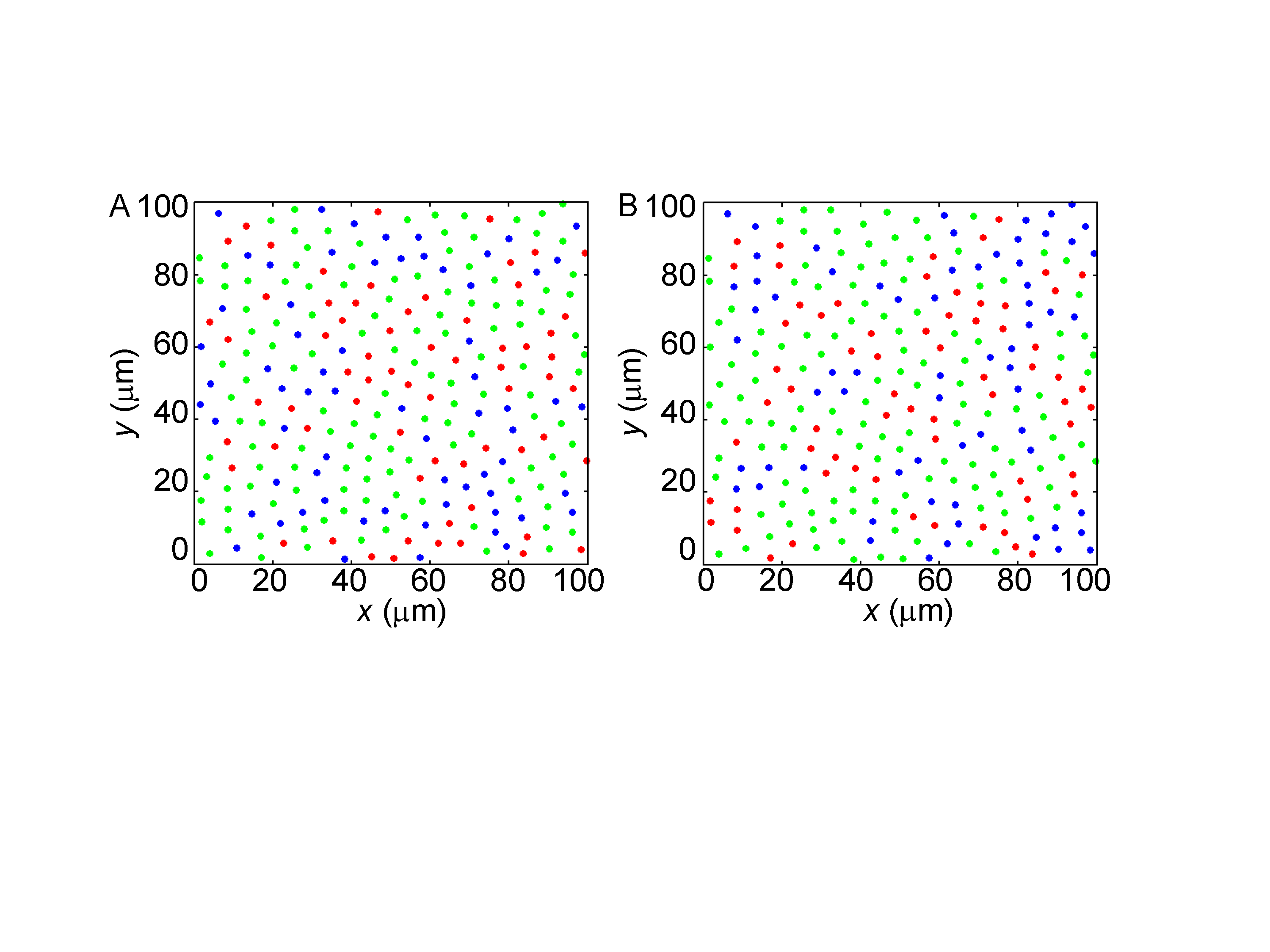

Supplement: Figure S4 — Simulated nuclear patterns of transcriptional states. Shown are examples of simulated fields exhibiting the transcriptional states of the nuclei, with (B) or without (A) inheritance. These two simulations were based on the exact nuclear lineage assignments shown in Figure S1. (TIFF) [file pone.0060876.s004.tiff]
