# Supplementary material for: A Spatial Point Pattern Analysis in Drosophila Blastoderm Embryos Evaluating the Potential Inheritance of Transcriptional States
Source: PLoS One. 2013 Apr 9;8(4):e60876. doi: 10.1371/journal.pone.0060876 (PMC3621909; doi:10.1371/journal.pone.0060876)
Supplement: Table S1 — Nuclear numbers of each type in the experimental fields of all 14 embryos. For each type of nuclei, the mean and the standard deviation among 14 embryos are given in the last column. (DOC) [file pone.0060876.s005.doc]

|  | #1 | #2 | #3 | #4 | #5 | #6 | #7 | #8 | #9 | #10 | #11 | #12 | #13 | #14 | µ±s.d. |
| --- | --- | --- | --- | --- | --- | --- | --- | --- | --- | --- | --- | --- | --- | --- | --- |
| Type 0 | 37 | 35 | 65 | 14 | 44 | 22 | 14 | 58 | 66 | 35 | 61 | 21 | 49 | 25 | 39±19 |
| Type 1 | 96 | 111 | 105 | 61 | 105 | 74 | 86 | 118 | 104 | 116 | 107 | 73 | 93 | 94 | 96±17 |
| Type 2 | 109 | 122 | 65 | 150 | 100 | 138 | 125 | 62 | 56 | 126 | 58 | 68 | 72 | 111 | 97±33 |
| Total | 242 | 268 | 235 | 225 | 249 | 234 | 225 | 238 | 226 | 277 | 226 | 162 | 214 | 230 | 232±37 |

**Table S1. Nuclear numbers of each type in the experimental field of all 14 embryos.**
